# Through Stronger Hindrance to Higher Reactivity: Influence of the Alkyl Chains on the Activation Energy of Ether Cleavage on Silicon

**DOI:** 10.1002/anie.202519990

**Published:** 2025-11-07

**Authors:** Timo Glaser, Gustav F. Nolte, Tamam Bohamud, Philip Keller, Mathieu G. Silly, Hendrik Weiske, Ralf Tonner‐Zech, Michael Dürr

**Affiliations:** ^1^ Institut für Angewandte Physik and Zentrum für Materialforschung Justus‐Liebig‐Universität Giessen Heinrich‐Buff‐Ring 16 35392 Giessen Germany; ^2^ Synchrotron SOLEIL L’Orme des Merisiers, Saint Aubin Gif sur Yvette 91192 France; ^3^ Wilhelm‐Ostwald‐Institut für Physikalische und Theoretische Chemie Universität Leipzig Linnéstr. 2 04103 Leipzig Germany

**Keywords:** Ab initio calculations, Nucleophilic substitution, Reaction mechanisms, Silicon, Surface chemistry

## Abstract

Reactivity in surface chemistry is often discussed in terms of the interaction between surface states and the functional groups of the reacting molecule. Herein, we demonstrate that for finite submonolayer coverage, i.e., surface coverage at which the molecular adsorbates cannot be treated as isolated molecules anymore, the seemingly innocent side chains of the adsorbate can also play a decisive role. For the example of ether cleavage on Si(001), which represents the surface analogue of an $S_{N}$2‐type reaction, we show both experimentally and based on ab initio calculations that steric hindrance by the side chains determines the activation energy for C‐O dissociation into the final state. In contrast to a simple expectation, the *stronger* steric hindrance of the butyl group in butyl methyl ether leads to a *lower* activation energy for ether cleavage on Si(001) when compared to diethyl ether. This effect was traced back to different degrees of destabilization of the precursor and the transition state. Given the fact that for almost all technologically relevant processes the surface coverage is typically above the isolated‐molecule limit, our findings are of general importance for surface functionalization and can help to properly design future experiments.

## Graphical abstract

For ether cleavage on Si(001), steric hindrance by the side chains was shown to determine the reactivity on the surface. In contrast to a simple expectation, the stronger steric hindrance of the butyl group in butyl methyl ether leads to a lower activation energy for ether cleavage on Si(001) when compared to diethyl ether. This effect was traced back to different degrees of destabilization of the precursor and the transition state.

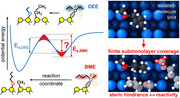

## Introduction

Semiconductor surface chemistry is the basis for the growth of semiconductor samples by means of deposition techniques such as chemical vapor deposition,^[^ ^1^, ^2^, ^3^ ^]^ molecular beam epitaxy,^[^ ^4^, ^5^ ^]^ or atomic layer deposition.^[^ ^3^, ^6^, ^7^, ^8^ ^]^ It is thus of great importance for technologies such as microelectronics and photovoltaics. Although the main concepts for the reactions of organic molecules on semiconductor surfaces, such as the technologically most important Si(001) surface, are well established,^[^ ^9^, ^10^, ^11^, ^12^ ^]^ previous studies focused on the influence of the different functional groups on the molecules' reactivity. Here, we show that not only the functional group itself but, surprisingly to the same extent, the seemingly innocent side groups can alter the reactivity of an organic molecule on a surface, as well. Moreover, we show that the change in reactivity on the surface can be opposite to the trend expected from the solution‐based counterparts and that the contribution of the different effects involved is difficult to judge on a qualitative basis.

**Figure 1:**
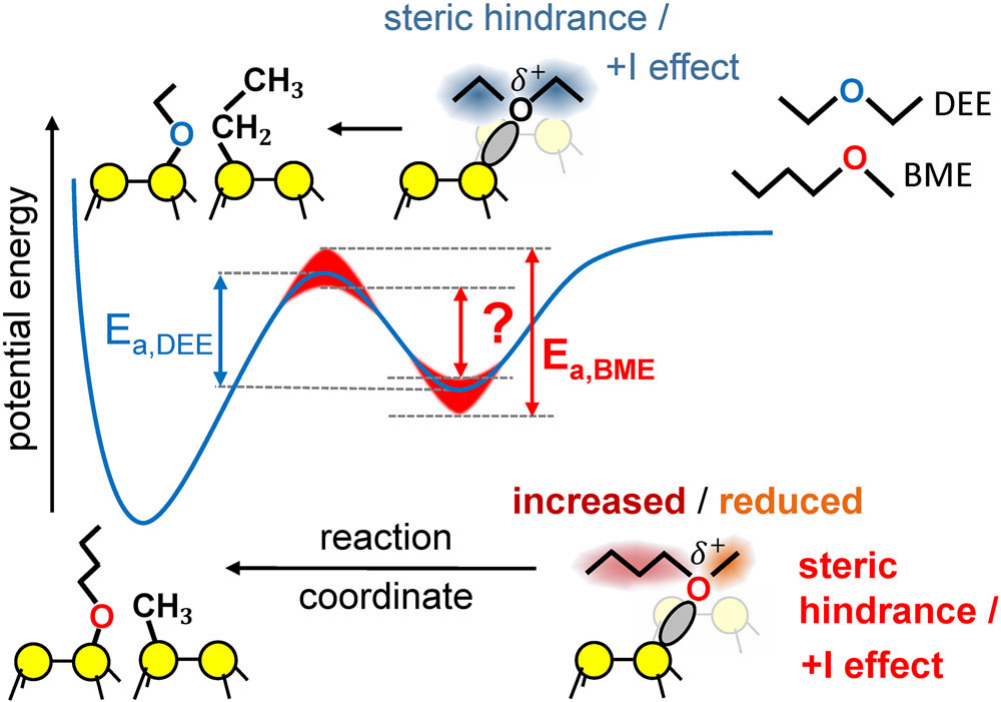
Schematic potential energy curve for BME and DEE on Si(001). Both ether molecules bind via a datively bound intermediate before they react via ether cleavage leading to two covalently bound fragments on the surface (one possible final configuration is illustrated for BME and DEE, respectively). Due to different strength of steric hindrance and the +I effect from the methyl and butyl groups compared to the ethyl groups, both a change in binding energy of the intermediate as well as a different height of the transition state energy and thus an in total different conversion barrier is expected for the two different molecules.

As a model system, we investigated ether cleavage on Si(001)^[^ ^13^, ^14^, ^15^ ^]^ which represents the surface analogue of an $S_{N}$2‐type reaction;^[^ ^16^ ^]^ in solution, the influence of effects such as the positive inductive (+I) effect as well as steric hindrance on $S_{N}$2‐type reactions are textbook knowledge. For ether cleavage on Si(001), we experimentally quantified the influence of these effects by determining the activation energy for the dissociation of butyl methyl ether (BME) and diethyl ether (DEE) on Si(001), which are only different in the length of their alkyl chains. Both ether molecules bind via a datively bound intermediate (precursor) to the surface before they react via ether cleavage leading to two covalently bound fragments (Figure 1).^[^ ^14^, ^16^ ^]^ In the intermediate, the oxygen atom donates electronic density into the empty Si dangling bond. This effect is in part compensated by the +I effect from the alkyl groups, stabilizing the intermediate state. A change in the strength of the +I effect might thus change the binding energy of the precursor and with this the conversion barrier. But also the transition state of the dissociation reaction as such can be influenced by a change of the +I effect (Figure 1), which might counteract the stabilization of the intermediate state with respect to its influence on the energy barrier and thus reactivity. In a similar way, steric hindrance at finite submonolayer surface coverage can destabilize the precursor as well as the transition state.

In order to address the question which of these effects is most relevant, we measured the energy barrier for ether cleavage of BME and DEE by means of real‐time X‐ray photoelectron spectroscopy (real‐time XPS) employing synchrotron radiation; a substantially lower energy barrier was deduced for BME/Si(001) when compared to DEE/Si(001). On the other hand, when we performed density functional theory (DFT)‐based calculations with various ether molecules with a systematic change of the length of the alkyl chains, a similar change of the stabilizing +I effect was found for both the precursor and transition state and thus no major change of the conversion barrier was observed. The experimental trend was only reproduced by the DFT calculations when taking into account a finite submonolayer surface coverage (as realized in the experiments) leading to different degrees of destabilization of the precursor and the transition state depending on the length of the alkyl chains involved.

## Results and Discussion

Prior to the real‐time XPS experiments, XPS spectra of BME and DEE in the intermediate ($T_{s}=90$ K) and in the covalently bound final state ($T_{s}=300$ K) were taken (Figure S1). In both, the intermediate as well as the final state, all peaks observed for BME and DEE are almost identical in position. Only in the C 1s spectra, the total intensity in the BME spectra is higher, which is explained by the additional carbon atom in the molecule. Indeed, for both temperatures, the additional intensity is found in the binding energy region of 285.0–286.0 eV, which is assigned to carbon atoms being bonded to other carbon or hydrogen atoms.^[^ ^17^, ^18^, ^19^ ^]^

**Figure 2:**
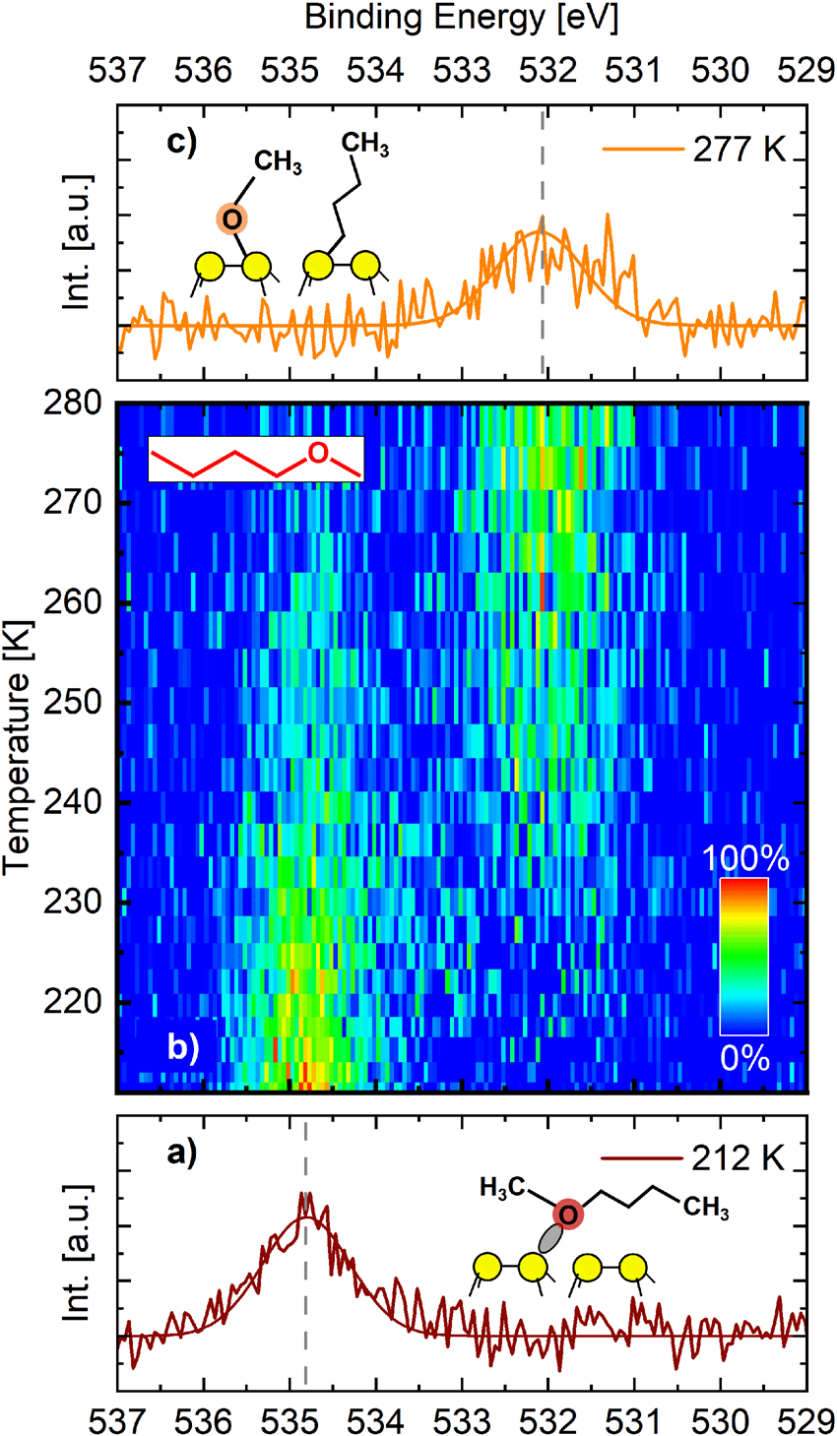
a) O 1s signal of BME on Si(001) at 212 K. b) O 1s signal change of BME on Si(001) while heating the sample from 210  to 280 K. With increasing temperature, the position of the O 1s line shifts toward lower binding energy, indicating ether dissociation from the intermediate state (datively bound oxygen atom) into the finale state (covalently bound oxygen). For each data line, the measurement time was the same (160 s) but the heating rate changed with temperature. c) O 1s signal of BME on Si(001) at 277 K.

We then made use of real‐time XPS measurements with synchrotron radiation to investigate the kinetics of the dissociation reaction that leads from the datively bonded intermediate to the covalently bonded final state. The measurements were performed both as a function of temperature and as a function of time at constant, elevated temperatures. In both cases, we made use of the O 1s signal: in the datively bound intermediate, the O 1s peak is found at 534.8 eV (Figure 2a).^[^ ^13^, ^14^ ^]^ After ether cleavage, it shifts to a binding energy of 532.1 eV (Figure 2c), which is associated with oxygen atoms covalently bound to the silicon surface.^[^ ^13^, ^14^, ^20^, ^21^ ^]^

In order to check for any effect of the synchrotron radiation itself on the reaction, the system was investigated at temperatures close to 210 K. At this temperature, no change of the O 1s peak assigned to the intermediate state was observed with increasing time; the obtained spectra did not differ in shape when compared to the spectra shown in Figure S1. The X‐ray intensity was thus concluded to be chosen low enough such that any reaction induced by the synchrotron radiation itself can be excluded.^[^ ^22^ ^]^

In a first real‐time experiment, XPS spectra were continuously recorded while the surface temperature was slowly increased. For the BME molecule, the result of this experiment is summarized in Figure 2b. Between 240 and 250 K, a clear shift of the intensity toward lower binding energy is observed, indicating the reaction into the final adsorption product.

**Figure 3:**
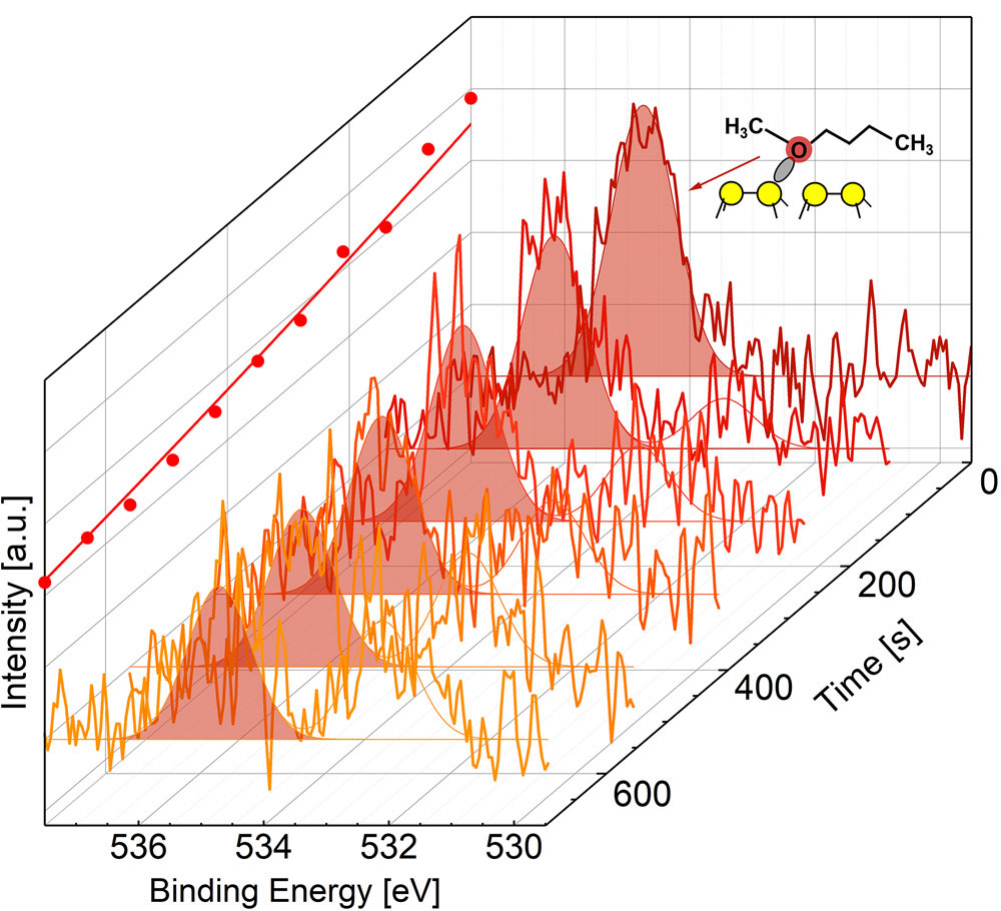
O 1s spectra of BME after adsorption on the Si(001) surface close to 210 K and rapid heating to 229 K. The time scale indicates the mean time span between the time the final temperature was reached and the time the respective spectrum was recorded. Spectra taken every 140 s are shown until 700 s. The peak at a binding energy of 534.8 eV is assigned to the datively bound oxygen of the intact BME molecule in the intermediate state on Si(001). With increasing time, the intensity shifts toward a peak at lower binding energy (532 eV), which is assigned to the covalently bound oxygen (Si‐**O**‐C) in the final ether cleavage product on the silicon surface.

In order to quantify the reaction rates, the O 1s spectra were recorded as a function of time at constant, elevated surface temperatures. In Figure 3, one of these isothermal experiments is illustrated by the O 1s spectra of BME on the Si(001) surface which were measured after different reaction times with the sample kept at $T_{s}=229$ K. We carried out 10 of these isothermal experiments for BME and 6 for DEE in the temperature range from 215  to 250 K. From the change of the O 1s line with time, e.g., from the decrease of signal intensity at 534.8 eV, i.e., the position of the O 1s line associated with the intact ether group in the datively bound intermediate state, the reaction rate for the given surface temperature was deduced.

**Figure 4:**
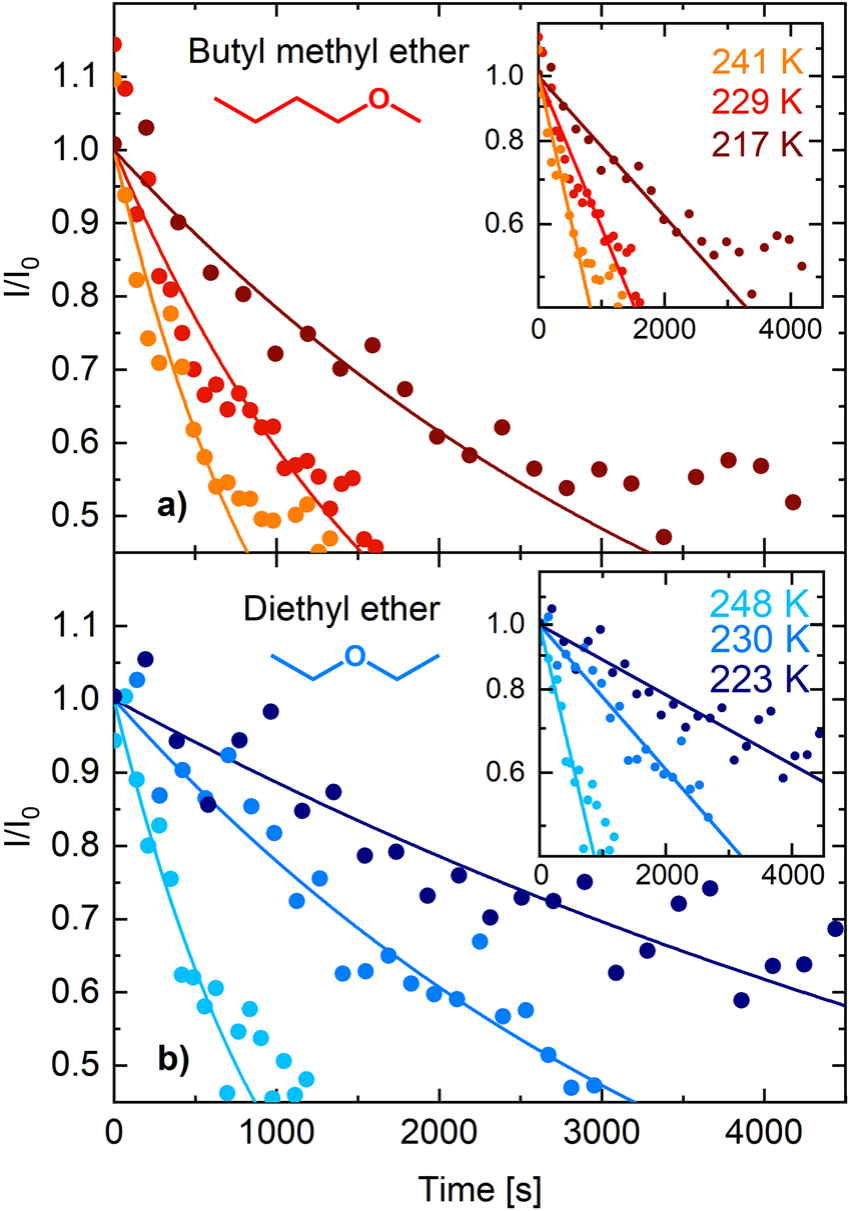
Signal intensity of the peak at 534.8 eV in the O 1s spectrum, which is assigned to oxygen involved in the dative O‐Si bond, measured as a function of time at several constant surface temperatures for a) BME/Si(001) and b) DEE/Si(001). The intensity I was normalized to the initial intensity $I_{0}$. An exponential fit to the data in the linear plot is shown for each temperature. A logarithmic plot of the same data and fits is shown in the two insets.

In Figure 4, this change in signal intensity of the peak at 534.8 eV is shown for three isothermal experiments (at three different temperatures as indicated) for each of the two molecules. It is obvious that the dissociation of BME proceeds faster when compared to DEE at similar surface temperature. For the initial decrease in signal, first order reaction kinetics were deduced from the logarithmic plots shown in the insets. Toward longer time scales, the signal saturates at a finite value indicating that not all of the ether molecules can be dissociated at the given temperature. This can be understood in terms of the finite submonolayer surface coverage: each reacted molecule occupies two dangling bonds instead of one (as it is the case for the intermediate state), thus reducing the number of available dangling bonds. As a consequence, for some of the adsorbed ether molecules, no (or only less reactive) reaction sites might be available once a larger share of the molecules have already reacted on the surface. Indeed, our DFT calculations (see below) point toward a strong influence of coverage on the reaction rate. In order to derive the rate constants of the ether cleavage reaction, the data of the first 800 s (for higher surface temperature) up to the first 2000 s (for lower surface temperature) have been employed.

**Figure 5:**
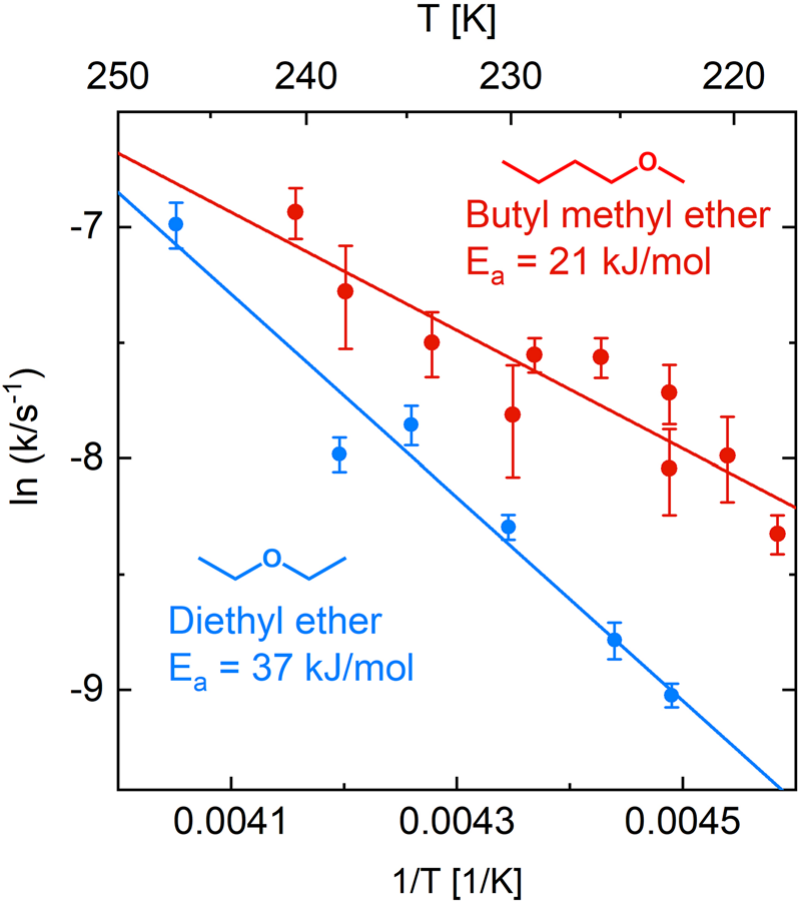
Arrhenius plot of the reaction constant of diethyl ether and butyl methyl ether. From the linear fit, an activation energy of 37 and 21 $\mathrm{kJ}\;{\mathrm{mol}}^{-1}$ was determined for diethyl ether and butyl methyl ether, respectively. Error bars of the single data points were deduced from the difference between data and fit functions in the isothermal reaction experiments, as shown in Figure 4.

The rate constants as deduced from the isothermal reaction experiments are summarized for both molecules in the Arrhenius plot shown in Figure 5. As we made use of the values of Reutzel et al.^[^ ^23^ ^]^ for DEE/Si(001) in order to calibrate the temperature in our experiment, we obtain the same activation energy (37±4 $\mathrm{kJ}\;{\mathrm{mol}}^{-1}$) and prefactor ($1\times 10^{4\pm 1}$ $s^{-1}$) for DEE as in Ref. [23]. For BME/Si(001), we then deduce an activation energy $E_{A}$ of 21 ±4 $\mathrm{kJ}\;{\mathrm{mol}}^{-1}$ and a prefactor A = 3.4 × 10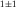 $s^{-1}$. Thus, although the molecules are most similar with respect to their molecular structure, the activation energy for ether cleavage of BME on Si(001) is substantially lower than for DEE on Si(001).

Before we concentrate on this difference in activation energy of the two molecules, we have a brief look on the pre‐exponential factors, which seem to be very low when compared to typically assumed values of $10^{12}\text{--}10^{13}$ $s^{-1}$. However, temperature dependent measurements of the kinetics of comparable systems are rare; an exception is the conversion of ethylene on Si(001) from the weakly bound intermediate into the covalently bound final adsorption state, for which a comparably low prefactor $A\approx 10^{2}$ $s^{-1}$ was determined.^[^ ^24^ ^]^ As outlined in Ref. [24], also in our experiments the low pre‐exponential factors can be rationalized by the high number of degrees of freedom of the intact molecules in the datively bonded intermediate state in contrast to the restricted geometry in the transition state.^[^ ^16^ ^]^ The former is associated with a large partition function $Z_{I}$, the latter with a strongly reduced partition function $Z_{‡}$. In the framework of transition state theory, the ratio between the partition functions, $Z_{‡}/Z_{I}$, is proportional to the pre‐exponential factor A; a large value of $Z_{I}$ thus results in a low value of A. For BME in the intermediate state, the number of the relevant degrees of freedom of the butyl chain is even higher than for the ethyl group in DEE, rationalizing the even lower value of A for BME.

**Figure 6:**
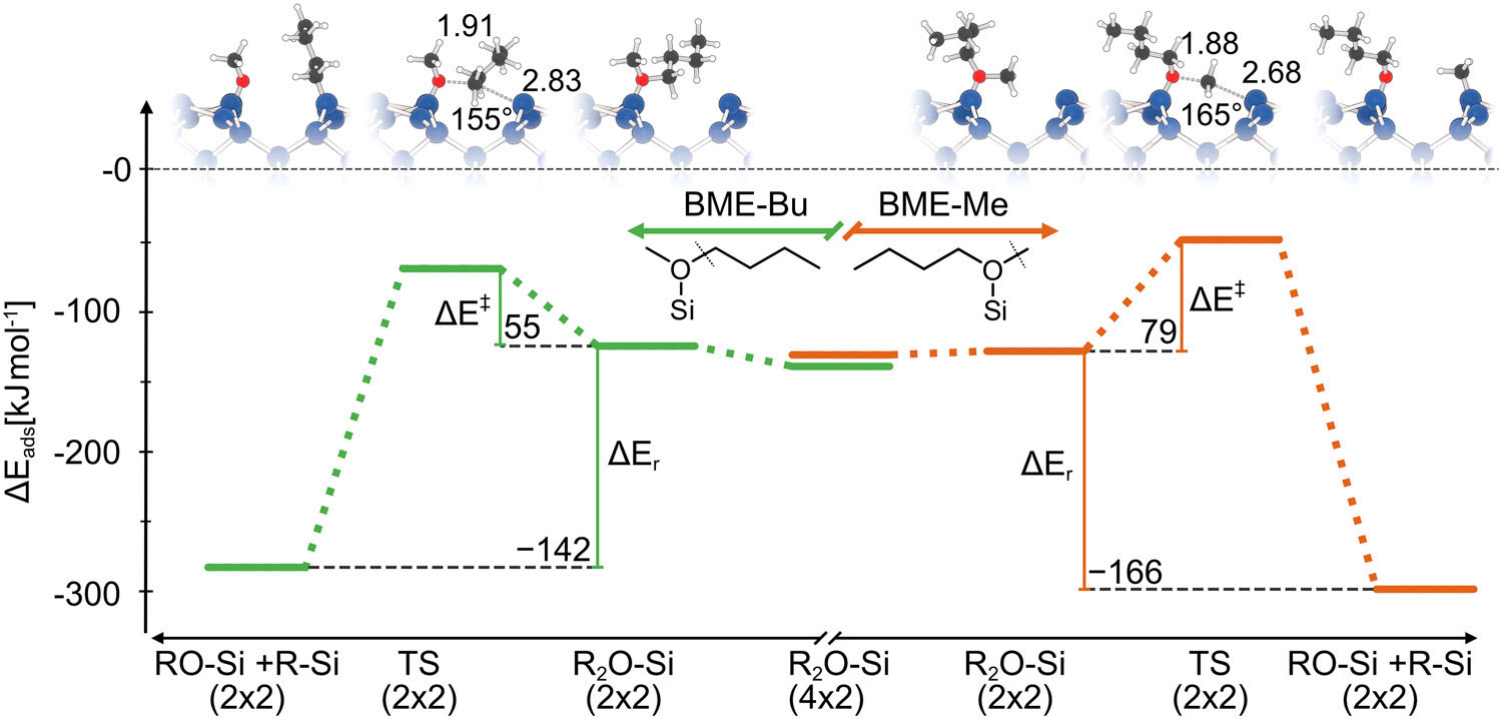
Reaction profile (PBE‐D3) for C‐O bond dissociation of BME from the datively bound precursor state ($R_{2}O$‐Si(2×2)) to the covalently bound final state (RO‐Si + R‐Si) stating the reaction barrier ($\Delta E^{‡}$) and the reaction energy ($\Delta E_{r}$) in $\mathrm{kJ}\;{\mathrm{mol}}^{-1}$. Left‐hand‐side denotes the butyl fission and right‐hand‐side the methyl fission as sketched in the ball‐and‐stick models on top; Si‐C and C‐O bond lengths (in Å) as well as the Si‐C‐O angle in the transition state are given.

We now come back to the overall difference in reactivity based on the different activation energies of BME and DEE. In order to identify the underlying physical origin, computational investigations were carried out. First, the influence of the +I effect on the relative barrier heights of BME and DEE dissociation was tested. The asymmetry of BME allows for the reaction to undergo a butyl or methyl fission, depending on the orientation of the molecule in the precursor state. As shown in Figure 6, different reaction barriers are found: 55 $\mathrm{kJ}\;{\mathrm{mol}}^{-1}$(breaking the $C_{\mathrm{butyl}}$‐O bond) and 79 $\mathrm{kJ}\;{\mathrm{mol}}^{-1}$(breaking the $C_{\mathrm{methyl}}$‐O bond). This is in line with the experimental data, for which the C 1s peak at 284.2 eV (Figure S1) indicates the silicon‐adsorbed butyl fragment whereas the methyl fragment would lead to a peak at 283.9 eV as deduced from reference experiments with methanol on Si(001).^[^ ^25^ ^]^ It further shows, in agreement with previous findings, that C‐O bond cleavage in such systems is under kinetic control.^[^ ^16^ ^]^

The difference between these reactions can be phenomenologically explained by the increased +I effect of the butyl group compared to the methyl group and thus an increased stability of the transition state. The quantitative analysis by computation (Table 1 and Figure 6) seemingly confirms this hypothesis. A stabilization of the transition state with respect to the reference energy of separated molecule and surface is found for the butyl path, as the adsorption energy for methyl fission was calculated to $\Delta E_{\mathrm{ads}}^{\mathrm{TS}}$ = −53$\;\mathrm{kJ}\;{\mathrm{mol}}^{-1}$ and for butyl fission to $\Delta E_{\mathrm{ads}}^{\mathrm{TS}}$ = −74$\;\mathrm{kJ}\;{\mathrm{mol}}^{-1}$.

**Table 1:** Computed energies (PBE‐D3) for DME, DEE, THF, BME‐Bu, and BME‐Me: Adsorption energies of the precursor state ($\Delta E_{\mathrm{ads}}^{P}$) and the transition state($\Delta E_{\mathrm{ads}}^{\mathrm{TS}}$) w. r. t. the well‐separated optimized molecule and surface; reaction barrier ($\Delta E^{‡}$), and reaction energy($\Delta E_{r}$) w. r. t. the precursor energy in the (2 × 2) surface reconstruction.

	DME	DEE	THF	BME‐Me	BME‐Bu
$\Delta E_{\mathrm{ads}}^{P}$	−104	−114	−131	−132	−129
$\Delta E_{\mathrm{ads}}^{\mathrm{TS}}$	−49	−57	−79	−53	−74
$\Delta E^{‡}$	56	57	51	79	55
$\Delta E_{r}$	−180	−158	−146	−166	−142

energies in $\mathrm{kJ}\;{\mathrm{mol}}^{-1}$

**Figure 7:**
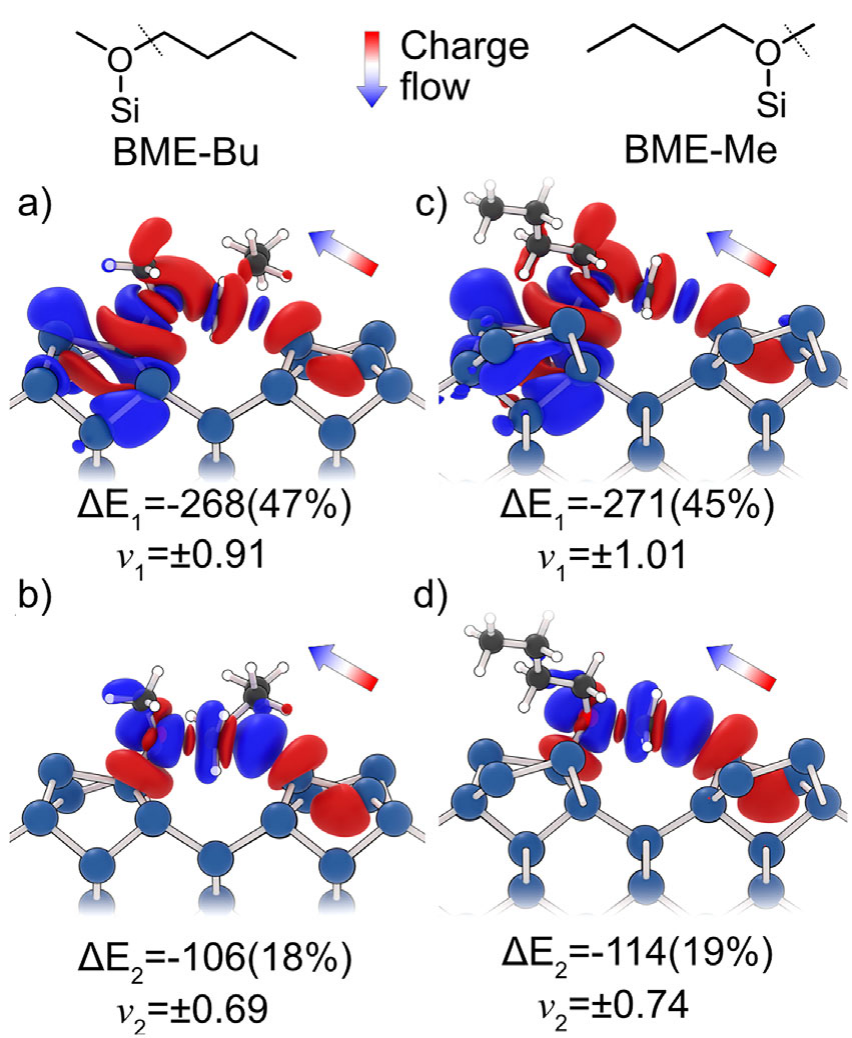
NOCV deformation densities obtained at PBE‐D3/TZ2P Γ‐only level. The two deformation densities with the largest contribution for the transition states for the butyl a,b) and methyl fission c,d) are shown. The charge flow is given from red to blue, corresponding to density depletion and accumulation, respectively. The contribution of the pairwise NOCV Eigenvalue pair ($\nu_{n}$) to $\Delta E_{\mathrm{orb}}$ is given in $\mathrm{kJ}\;{\mathrm{mol}}^{-1}$ and as percentage value of the total orbital interaction energy (see Supporting Information).

To shed more light on the underlying electronic factors, the chemical bond was analyzed at the transition state using the energy decomposition analysis for extended systems (pEDA analysis, details see Supporting Information) and the Natural Orbitals for Chemical Valence (NOCV) extension (Figure 7). The obtained data agree well with the literature‐known behavior of the S_N_2‐like reaction of ethers on Si(001):^[^ ^16^ ^]^ We see charge flow from the neighboring silicon atom (red lobe in Figure 7a,c) attacking the C‐O bond from the back and breaking of the C‐O bond (blue lobes between C and O atoms in Figure 7b,d) at the same time. However, the +I‐effect cannot be confirmed by this quantitative analysis. The expectation would have been that the attractive interaction terms ($E_{\mathrm{orb}}$, $E_{\mathrm{elstat}}$) are stronger for the butyl fission. However, the results are very similar for both reaction pathways (see Table S1). Instead, the picture is more complex. The interaction energy for the TS of the butyl fission is indeed larger ($\Delta \Delta E_{\mathrm{int}}=-18\;\mathrm{kJ}\;{\mathrm{mol}}^{-1}$), in line with the larger adsorption energy. However, this is mainly due to an increase in dispersion attraction ($\Delta \Delta E_{\mathrm{disp}}=-12\;\mathrm{kJ}\;{\mathrm{mol}}^{-1}$) and a decrease of Pauli repulsion ($\Delta \Delta E_{\mathrm{Pauli}}=-28\;\mathrm{kJ}\;{\mathrm{mol}}^{-1}$). The orbital interaction is even lower compared to the TS for the methyl fission ($\Delta \Delta E_{\mathrm{orb}}=+22\;\mathrm{kJ}\;{\mathrm{mol}}^{-1}$) and the electrostatic attraction stays the same ($\Delta \Delta E_{\mathrm{elstat}}=0\;\mathrm{kJ}\;{\mathrm{mol}}^{-1}$).

We put these findings in perspective by computations of a set of ether molecules with different alkyl chain lengths: dimethyl ether (DME), diethyl ether (DEE),^[^ ^16^ ^]^ and tetrahydrofuran (THF)^[^ ^16^ ^]^ (Table 1). From the concept of the +I effect, we would expect the reaction barrier to decrease in the following order: DME = BME‐Me > DEE > THF > BME‐Bu. However, the quantitative analysis does not show this trend. DME has a lower reaction barrier compared to that of the methyl fission in BME (BME‐ME) and is comparable to that in DEE and BME‐Bu. The ring strain in THF, even if it is small, might lead to the lowest reaction barrier in the series as a strained ring is more easily opened by an attacking nucleophile. This is reflected in a lower preparation energy ($\Delta E_{\mathrm{prep}}$ by pEDA analysis; for details see Supporting Information). In the precursor adsorption energy ($\Delta E_{\mathrm{ads}}^{P}$), no clear trend arises, either. Only for the stabilization of the transition state structures ($\Delta E_{\mathrm{ads}}^{\mathrm{TS}}$) of the acyclic ethers, the expected trend is found: The energies increase with increasing chain length, with THF being additionally stabilized due to the geometric effect.^[^ ^16^ ^]^

**Figure 8:**
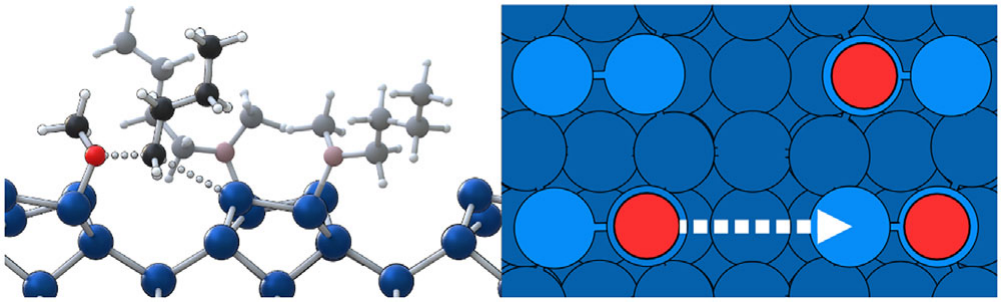
Right: Schematic representation of the reaction investigated at finite submonolayer surface coverage. The silicon dimers are represented in light blue, the lower silicon layers are shown in darker blue; the red circles show the adsorption sites of the datively bonded oxygen atoms. The silicon slab is covered with the respective ether molecules and one of the molecules is reacting with the dimer of the neighboring dimer row (white dashed arrow). On the left, the respective transition state structure is shown.

**Figure 9:**
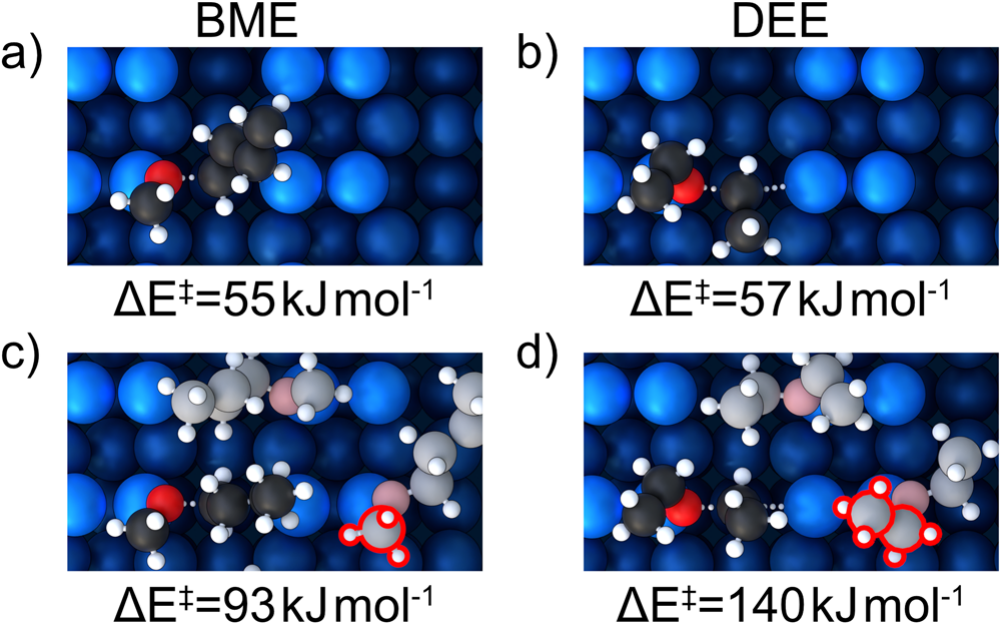
Calculated reaction barriers (PBE‐D3) of the butyl fission of BME a,c) and the reaction of DEE b,d) in $\mathrm{kJ}\;{\mathrm{mol}}^{-1}$  on clean Si(001) a,b) and on the surfaces with increased coverage c,d). The methyl/ethyl group on the opposing silicon dimer are labeled in red in (c) and (d), respectively.

To understand the discrepancy between the calculated barriers and the experimentally determined activation energies (Figure 5), the single‐molecule study was extended to finite submonolayer coverage (Figure 8). The reaction of one ether molecule on a surface where 3 out of 4 dimers were covered with intact molecules was considered (Figures 8 and 9). This arrangement deliberately exceeds the experimental coverage to better identify the effect of increased surface coverage. The chosen adsorption configuration representatively shows the steric and electronic effects of surrounding adsorbates. Of course, many more configurations and conformations could be considered. However, the goal is to show the general effect of increased coverage on the reaction barrier for a representative configuration. As expected, a destabilization of the precursor state occurs due to the steric repulsion of the additional molecules in the direct neighborhood (which are also in the precursor state). However, also the transition state is destabilized, leading to an increase of the reaction barrier for BME to $\Delta E=95\;\mathrm{kJ}\;{\mathrm{mol}}^{-1}$. For DEE, however, this increase is even more significant, resulting in a barrier of $\Delta E=142\;\mathrm{kJ}\;{\mathrm{mol}}^{-1}$. This large difference is the result of a higher precursor destabilization for BME and a larger transition state destabilization for DEE (compare Table S1).

This finding supports the experimental evidence much more convincingly than in the single‐molecular case. The destabilization effect on the intact ether molecules in the precursor state acts, as expected, more strongly on the long butyl chain of BME than on DEE. However, in the transition state, the ether molecule on the opposite dimer plays a more important role. Here, the methyl group on the BME molecule shows to be far less repulsive than the ethyl group of DEE. A similar effect has recently been observed in the context of thin‐film deposition (coined ”reactivity reduction“) at high coverage.^[^ ^26^ ^]^ For our system, in combination with the stronger destabilization of the precursor state by the butyl group, it leads to the *increase* of reactivity of BME when compared to DEE. Thus, our experimental determination of the activation barriers for ether cleavage on Si(001) in combination with the DFT calculations clearly demonstrate the importance of *all* aspects of destabilization by the alkyl side chains on surface reactivity at finite submonolayer surface coverage.

We have to note that the calculated transition state energies for ether cleavage at the chosen coverage are positive with respect to the molecule in the gas phase. In the calculated situation, desorption would thus be preferred over dissociation. However, for the calculated configuration, the very local coverage, i.e., taking into account the two dimers directly involved in the reaction, is $\Theta_{\mathrm{local}}=1$. We thus expect the same effects to be operative, but with reduced strength, at medium coverage as realized in the experiments, leading to the same trend.

## Conclusion

In conclusion, we could experimentally show that the length of the alkyl chains has a strong effect on the reactivity of ether cleavage on Si(001). Although the system represents a surface analogue of an $S_{N}$2‐type reaction, the observed trends caused by steric hindrance and the +I effect are more complex than for the gas phase or solution‐based reactions. In particular, in the DFT calculations the change in the +I effect was not sufficient to explain the experimentally observed difference in the activation energy for BME and DEE dissociation. Only when taking into account a finite submonolayer coverage, the experimentally observed trend could be reproduced, taking into account both the destabilization of the precursor and the transition state by steric hindrance of the alkyl side chains. The results of the study thus show that the coverage can have a significant impact on the observed energy landscape of the reaction and that the molecular geometry, in particular of the non‐reactive side chains, plays a decisive role on how the coverage will affect the reaction barrier. Our results are thus of importance for a realistic modeling of surface reactions as they show that an approach beyond the isolated‐molecule limit is mandatory, e.g., for growth processes.

## Conflict of Interests

The authors declare no conflict of interest.

## Supporting information

Supporting Information

## Data Availability

The data that support the findings of this study are available from the corresponding author upon reasonable request.
