# Supplementary material for: Through Stronger Hindrance to Higher Reactivity: Influence of the Alkyl Chains on the Activation Energy of Ether Cleavage on Silicon
Source: Angew Chem Int Ed Engl. 2025 Nov 7;65(1):e19990. doi: 10.1002/anie.202519990 (PMC12759237; doi:10.1002/anie.202519990)
Supplement: Supplementary file 1 — Supporting Information [file ANIE-65-e19990-s001.pdf]

Supporting Information for:

Through Stronger Hindrance to Higher Reactivity: Influence of the Alkyl Chains on the Activation Energy of Ether Cleavage on Silicon

Timo Glaser,† Gustav F. Nolte,† Tamam Bohamud,† Philip Keller,† Mathieu G. Silly,‡ Hendrik Weiske,¶ Ralf Tonner-Zech,*,¶ and Michael Dürr*,§

†*Institut für Angewandte Physik and Zentrum für Materialforschung, Justus-Liebig-Universität Giessen, Heinrich-Buff-Ring 16, 35392 Giessen, Germany*

‡*Synchrotron SOLEIL, L'Orme des Merisiers, Saint Aubin, Gif sur Yvette 91192, France*

¶*Wilhelm-Ostwald-Institut für Physikalische und Theoretische Chemie, Universität Leipzig, Linnéstr. 2, 04103 Leipzig, Germany*

§*Institut für Angewandte Physik and Zentrum für Materialforschung, Justus-Liebig-Universität Giessen, Heinrich-Buff-Ring 16, D-35392 Giessen, Germany*

E-mail: ralf.tonner@uni-leipzig.de; michael.duerr@ap.physik.uni-giessen.de

This Supporting Information includes

(I) Details of the experimental and calculational methods used

(II) XPS data on BME and DEE on Si(001) measured at 90 K and 300 K

(III) Details of the DFT calculations

(I) Details of the experimental and calculational methods used

The experiments were conducted in ultrahigh vacuum (UHV) with a base pressure $< 5 \times 10^{-10}$ mbar at TEMPO beamline, SOLEIL synchrotron (Gif sur Yvette, France).[1] Si(001) samples were prepared by degassing at 700 K and repeated direct current heating cycles to above 1450 K. By cooling rates of about 1 K/s, a well ordered 2×1 reconstruction is obtained.[2,3] Prior to the adsorption and reaction experiments, sample cleanliness was verified by means of XPS measurements. A small signal was observed in the O 1s spectra, which was attributed to the adsorption of water and subtracted from the following spectra. Diethyl ether (DEE) and butyl methyl ether (BME) were dosed via a UHV compatible leak-valve from the vapor phase in a test tube. Both molecules were purchased from Sigma Aldrich (purity 99 %). During adsorption, which took place at $T_s \approx 210$ K, the sample was positioned with its surface in front of the gas inlet. A surface coverage of about half of a monolayer (1 ML equals one ether molecule per Si dimer) was typically adsorbed as a trade-off between signal intensity and a sufficient number of dangling bonds available for the further reaction steps. After adsorption of the molecules, the surface temperature was increased via direct current heating of the Si sample and XPS spectra were recorded in real time. For both molecules, two different experiments were performed: First, the sample current was increased from 0 A up to 0.5 A by ramping the current with 0.001 mA/s resulting in an overall temperature range of 210 to 350 K. Second, isothermal experiments at different constant surface temperatures were performed by a fast ramp to a given sample current for each adsorption experiment. Temperature calibration was carried out by referencing the rate constants measured for the dissociation of DEE to the values reported in literature for this reaction.[4] We note that the

experiments in Ref. 4 were performed at lower coverage compared to this study. This might influence the absolute values of the experimentally determined activation energies (see main paper) but does not affect the relation between $E_{\mathrm{A,DEE}}$ and $E_{\mathrm{A,BME}}$

For the real-time XPS experiments, a HU80 Apple II undulator photon source was set to deliver linearly polarized light. The end-station at the TEMPO beamline was fitted with a modified MBS A-1 photoelectron analyzer.[1,5] The angle of the incoming synchrotron beam was 44°, and the electron takeoff angle was 0° with respect to the surface normal. In order to reduce the beam intensity and thus avoid beam induced reactions or damage, the beam was defocused before entering the sample chamber, resulting in a spot of $\approx 500\ \mu$m in diameter [full witdh at half maximum (fwhm)]. The photoemission spectra were taken at a photon energy of hν = 700 eV. The overall energy resolution was set to 170 meV, the electron analyzer was operated in sweep mode with adjusted energy windows for each binding energy (BE) region (O 1s, C 1s, and Si 2p). Depending on the width of the energy window, 40 to 70 s were necessary to acquire the spectrum of one binding energy region. All XPS spectra were referenced to the Si $2p_{3/2}$ bulk peak at a binding energy of 99.4 eV[6] in order to compensate possible line shifts due to the voltage drop across the sample when heating the surface by means of direct current heating. The lines were fitted with Voigt profiles with the fwhm set to 1.1 to 1.2 eV. Additional XPS measurements at a surface temperature of 90 K and 300 K were performed in a separate experimental set-up using an Al K_α X-ray source with a monochromator (Omicron XM1000) and a hemispherical energy analyser (Omicron EA125) (compare Fig. S1).

Structural optimizations have been carried out using density functional theory (DFT) with periodic boundary conditions as implemented in the Vienna ab initio simulation package (VASP) version 5.4.4.[7–10] The exchange correlation functional of Perdew, Burke and Enzerhof (PBE)[11] along with the semi-empirical D3 dispersion correction with Becke-Johnson damping was used.[12,13] VASP's projector augmented wave (PAW)[14,15] formalism was employed with a kinetic energy cutoff of 400 eV. A Γ(221)-centered Monkhorst-Pack grid was

utilized for sampling reciprocal space. The convergence criteria chosen for energies and forces were 10^{-6} eV and 10^{-2} eV Å^{-1}, respectively. The energy decomposition analysis for extended systems(pEDA)[16] has been carried out with AMS-BAND,[17,18] using Slater-type orbitals,[19] and a Becke 3 grid is set for integration.[20] The calculations were performed at PBE-D3(BJ)/TZ2P level with a (33) regular Monkhorst-Pack grid used in the pEDA calculations, but only (11) for the natural orbitals for chemical valence (NOCV). All computational raw data are found in the Zenodo repository with DOI 10.5281/zenodo.17416835.

(II) XPS data on BME and DEE on Si(001) measured at 90 K and 300 K

Prior to the real-time XPS experiments, XPS spectra of BME and DEE in the intermediate (T_s = 90 K) and in the covalently bound state (at room temperature) were taken (Fig. S1). The O 1s spectra show almost identical peak positions for DEE and BME on Si(001): In case of adsorption at 90 K (Fig. S1(a), left side), a major peak at 534.8 to 534.9 eV is observed, which is assigned to the datively bound oxygen atom in the intermediate of DEE and BME, respectively.[4,21,22] In case of adsorption at 300 K (Fig. S1(b), left side), one single peak at a binding energy of 532.1 eV is observed, which is assigned to oxygen atoms covalently bound to the silicon surface.[21–24] This bonding situation is found in the reaction products of both DEE and BME on Si(001). In the C 1s spectra, the peak positions in the spectra of BME and DEE are almost identical as well. However, both in case of adsorption at 90 K as well as in case of adsorption at 300 K, the total intensity in the BME spectra is higher, which is explained by the additional carbon atom in the molecule. Indeed, for both temperatures, the additional intensity is found in the binding energy (BE) region of 285.0 to 286.0 eV (grey area in the spectra), which is assigned to carbon atoms binding with other carbon or hydrogen atoms.[25–27] In the spectra taken after adsorption at 90 K (Fig. S1(a), right side), in addition to the peak at 285.8 eV, a peak at higher BE (287.5 eV) is observed. This peak is assigned to carbon atoms binding to the oxygen atom forming the dative bond in the intermediate of BME and DEE on the silicon surface (**C**-O-Si).[22] The intensity ratio of the two peaks is 1:1 for DEE and 2:3 for BME, in accordance with

their chemical structure. When the molecules are adsorbed at 300 K, the C 1s spectrum can be decomposed into three components (Fig. S1(b), right side). The peak at a binding energy of 284.2 eV is assigned to carbon atoms binding to the silicon surface (**C**-Si),[22,25,27] the peak around 285.3 eV to carbon atoms binding to other carbon or hydrogen atoms.[25–27] The peak at 286.5 eV is assigned to carbon atoms bound to one oxygen atom (C-**C**-O).[25] The three peak positions and relative intensities (1:2:1 for DEE and 1:3:1 for BME) are thus in very good agreement with the values expected after ether cleavage of DEE and BME on the Si(001) surface.

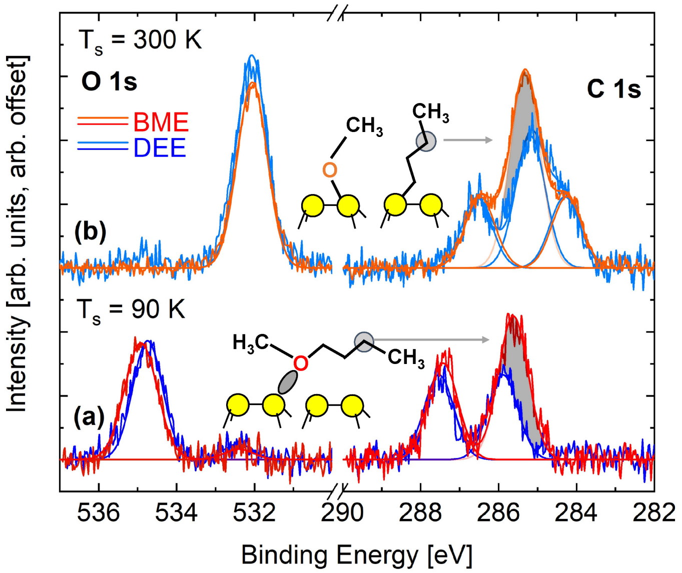

Figure S1: O 1s and C 1s spectra of DEE and BME of ≈ 0.5 to 1 ML on Si(001) after adsorption at 90 K (a) and 300 K (b). The spectra are scaled in intensity for better comparison. (a) The peaks in the O 1s spectrum at a binding energy of 534.8 to 534.9 eV are assigned to the datively bound oxygen atom of the ether in the intermediate state on Si(001). A minor contribution of covalently bound species, e.g., at minority sites such as steps or defects, is indicated by the small peak at 532.3 eV. In the C 1s spectrum, for both molecules the same two peaks are observed. In case of BME, the intensity of the peak at lower binding energy is more intense (difference shown as grey area) as a consequence of the additional carbon atom in the molecule. (b) In the O 1s spectrum, one peak at a binding energy of 532.1 eV is observed, which is assigned to the O-Si configuration on the surface as a result of ether cleavage. In the C 1s spectrum, three peaks are observed, but in the case of BME, the peak around 285.5 eV is more intense (grey area) as a result of the additional carbon atom in the molecule.

(III) Details of the DFT calculations

The pEDA (Tab. S1) shows larger interaction energy for the transition state of the butyl fission in general, with -264 $\mathrm{kJ\,mol^{-1}}$ compared to -246 $\mathrm{kJ\,mol^{-1}}$. However, a deduction of the +I effect is not possible due to overlapping effects in the interaction of the molecule and surface fragments. The main differences between the methyl and butyl fission shows to be a larger dispersion interaction and a smaller Pauli repulsion, both not resulting from the +I effect, but the more favorable position of the alkyl chain in the trench, compared to the dimer row in the methyl fission.

Table S1: pEDA results at PBE-D3(BJ) level for the transition states of the methyl (BME-Me) and butyl (BME-Bu) fission. [a]Contributions to ΔE_{int}; [b]attractive contributions of ΔE_{int}(elec); [c]bonding energy obtained with PBE-D3(BJ)/PAW and cutoff of 400 eV.

	BME-Me		BME-Bu	
ΔE_{int}	-246		-264	
ΔE_{int}(disp)	-62	(25%)[a]	-74	(28%)[a]
ΔE_{int}(elec)	-184	(75%)[a]	-190	(72%)[a]
$\Delta E_{\mathrm{Pauli}}$	1028		1000	
$\Delta E_{\mathrm{elstat}}$	-614	(51%)[b]	-614	(52%)[b]
ΔE_{orb}	-598	(49%)[b]	-576	(48%)[b]
ΔE_{prep}(mol)	145		151	
ΔE_{prep}(surf)	44		36	
ΔE_{prep}	189		186	
ΔE_{bond}	-57	(-54)[c]	-78	(-74)[c]

energies in $\mathrm{kJ\,mol^{-1}}$

The pEDA results for ethers with alkyl chains of different length show a similar picture as well (Tab. S2). The interaction energy decreases with increasing chain length for the acyclic ethers, with the dispersion interaction dominating the trend. The decreased reaction barrier for THF can be explained using the preparation energy, resembling a lower strain due to the cyclic structure, compared to all other ethers.

Table S2: pEDA results at PBE-D3(BJ) level for the transition states of ether cleavage of DME, DEE, and THF. [a]Contributions to ΔE_{int}; [b]attractive contributions of $\Delta E_{\mathrm{int}}(\mathrm{elec})$; [c]bonding energy obtained with PBE-D3(BJ)/PAW and cutoff of 400 eV.

	DME		DEE		THF	
ΔE_{int}	−227		−254		−240	
$\Delta E_{\mathrm{int}}(\mathrm{disp})$	−40	(18%)[a]	−58	(23%)[a]	−50	(25%)[ba]
$\Delta E_{\mathrm{int}}(\mathrm{elec})$	−187	(82%)[a]	−196	(77%)[a]	−190	(75%)[a]
$\Delta E_{\mathrm{Pauli}}$	960		1002		976	
$\Delta E_{\mathrm{elstat}}$	−582	(51%)[b]	−611	(51%)[b]	−597	(51%)[b]
ΔE_{orb}	−566	(49%)[c]	−586	(49%)[b]	−568	(49%)[b]
$\Delta E_{\mathrm{prep}}(\mathrm{mol})$	142		161		128	
$\Delta E_{\mathrm{prep}}(\mathrm{surf})$	35		35		32	
ΔE_{prep}	178		196		160	
ΔE_{bond}	−49	(-49)[c]	−58	(-57)[c]	−81	(-79)[c]

energies in kJ mol^{-1}

For the study of higher coverages, the pristine silicon slab was precovered using two ether molecules. Therefore, the adsorption energy is calculated as follows:

$$\Delta E_{\mathrm{ads}}^{\mathrm{cov}} = E_{\mathrm{full}} - E_{\mathrm{precovered}} - E_{\mathrm{mol}} \tag{1}$$

according to the precovered state and full-covered state in Fig. S2.

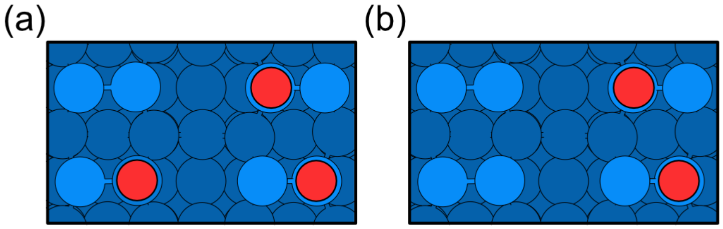

Figure S2: Full-coverage configuration (a) and precovered configuration (b) for the calculation of adsorption energies $\Delta E_{\mathrm{ads}}^{cov}$

Considered was only the reaction of an additional ether molecule, while the other ether molecules stay intact. The reaction barrier of BME is significantly smaller than that of DEE (Tab. S3). This is the consequence of a larger precursor destabilization for BME than for DEE, but a less destabilized transition state for BME.

Table S3: Study of coverage effects at PBE-D3(BJ) level. Calculated are the adsorption energies $\Delta E_{\mathrm{ads}}^{\mathrm{cov}}$ (according to eq. 1) for the precursor (P) and transition state (TS) as well as the resulting reaction barrier $\Delta E^{\ddagger}$ and energy ΔE_{r}.

	BME-Bu	DEE
$\Delta \mathrm{E}_{\mathrm{ads}}^{\mathrm{cov}}$(P)	-51	-88
$\Delta \mathrm{E}_{\mathrm{ads}}^{\mathrm{cov}}$(TS)	42	52
$\Delta \mathrm{E}^{\ddagger}$	95	142
$\Delta \mathrm{E}_{\mathrm{r}}$	-217	-164

energies in $\mathrm{kJ\,mol^{-1}}$

References

(1) Polack, F.; Silly, M.; Chauvet, C.; Lagarde, B.; Bergeard, N.; Izquierdo, M.; Chubar, O.; Krizmancic, D.; Ribbens, M.; Duval, J. P.; Basset, C.; Kubsky, S.; Sirotti, F. TEMPO: a New Insertion Device Beamline at SOLEIL for Time Resolved Photoelectron Spectroscopy Experiments on Solids and Interfaces. *AIP Conf. Proc.* **2010**, *1234*, 185 – 190.

(2) Schwalb, C. H.; Lawrenz, M.; Dürr, M.; Höfer, U. Real-space investigation of fast diffusion of hydrogen on Si(001) by a combination of nanosecond laser heating and STM. *Phys. Rev. B* **2007**, *75*, 085439.

(3) Mette, G.; Schwalb, C.; Dürr, M.; Höfer, U. Site-selective reactivity of ethylene on clean and hydrogen precovered Si(001). *Chem. Phys. Lett.* **2009**, *483*, 209 – 213.

(4) Reutzel, M.; Lipponer, M.; Dürr, M.; Höfer, U. Binding Energy and Dissociation Barrier: Experimental Determination of the Key Parameters of the Potential Energy Curve of Diethyl Ether on Si(001). *J. Phys. Chem. Lett.* **2015**, *6*, 3971–75.

(5) Glaser, T.; Länger, C.; Heep, J.; Meinecke, J.; Silly, M. G.; Koert, U.; Dürr, M. Starting from a Fixed Geometry: Real-Time XPS Investigation of a Surface Reaction with Controlled Molecular Configurations. *J. Phys. Chem. C* **2020**, *124*, 22619–22624.

(6) Liu, H.; Hamers, R. J. An X-ray photoelectron spectroscopy study of the bonding of unsaturated organic molecules to the Si(001) surface. *Surf. Sci.* **1998**, *416*, 354–62.

(7) Kresse, G.; Hafner, J. Ab initio molecular dynamics for liquid metals. *Phys. Rev. B* **1993**, *47*, 558–561.

(8) Kresse, G.; Hafner, J. Ab initio molecular-dynamics simulation of the liquid-metal–amorphous-semiconductor transition in germanium. *Phys. Rev. B* **1994**, *49*, 14251–14269.

(9) Kresse, G.; Furthmüller, J. Efficiency of ab-initio total energy calculations for metals and semiconductors using a plane-wave basis set. *Comp. Mat. Sci.* **1996**, *6*.

(10) Kresse, G.; Furthmüller, J. Efficient iterative schemes for ab initio total-energy calculations using a plane-wave basis set. *Phys. Rev. B* **1996**, *54*, 11169–11186.

(11) Perdew, J. P.; Burke, K.; Ernzerhof, M. Generalized Gradient Approximation Made Simple. *Phys. Rev. Lett.* **1996**, *77*, 3865–3868.

(12) Grimme, S.; Antony, J.; Ehrlich, S.; Krieg, H. A consistent and accurate ab initio parametrization of density functional dispersion correction (DFT-D) for the 94 elements H-Pu. *J. Chem. Phys.* **2010**, *132*, 154104.

(13) Grimme, S.; Ehrlich, S.; Goerigk, L. Effect of the damping function in dispersion corrected density functional theory. *J. Comput. Chem.* **2011**, *32*, 1456–1465.

(14) Kresse, G.; Joubert, D. From ultrasoft pseudopotentials to the projector augmented-wave method. *Phys. Rev. B* **1999**, *59*, 1758–1775.

(15) Blöchl, P. E. Projector augmented-wave method. *Phys. Rev. B* **1994**, *50*, 17953–17979.

(16) Raupach, M.; Tonner, R. A periodic Energy Decomposition Analysis (pEDA) method for the Investigation of Chemical Bonding in Extended Systems. *J. Chem. Phys* **2015**, *142*.

(17) te, V. G.; Baerends, Precise density-functional method for periodic structures. *Phys. Rev. B Condens. Matter* **1991**, *44*, 7888–7903.

(18) BAND 2022.1. SCM, Theoretical Chemistry, Vrije Universiteit, Amsterdam, The Netherlands, http://www.scm.com.

(19) Van Lenthe, E.; Baerends, E. J. Optimized Slater-type basis sets for the elements 1-118. *J. Comput. Chem.* **2003**, *24*, 1142–1156.

(20) Franchini, M.; Philipsen, P. H. T.; Visscher, L. The Becke Fuzzy Cells Integration Scheme in the Amsterdam Density Functional Program Suite. *J. Comput. Chem.* **2013**, *34*, 1819–1827.

(21) Mette, G.; Reutzel, M.; Bartholomäus, R.; Laref, S.; Tonner, R.; Dürr, M.; Koert, U.; Höfer, U. Complex Surface Chemistry of an Otherwise Inert Solvent Molecule: Tetrahydrofuran on Si(001). *Chem. Phys. Chem.* **2014**, *15*, 3725–3728.

(22) Reutzel, M.; Mette, G.; Stromberger, P.; Koert, U.; Dürr, M.; Höfer, U. Dissociative Adsorption of Diethyl Ether on Si(001) Studied by Means of Scanning Tunneling Microscopy and Photoelectron Spectroscopy. *J. Phys. Chem. C* **2015**, *119*, 6018–23.

(23) Hwang, H.-N.; Baik, J. Y.; An, K.-S.; Lee, S. S.; Kim, Y. Selectivity of the Chemisorption of Vinylacetic Acid on the Si(001) 2×1 Surface. *J. Phys. Chem. B* **2004**, *108*, 8379 – 8384.

(24) Länger, C.; Bohamud, T.; Heep, J.; Glaser, T.; Reutzel, M.; Höfer, U.; Dürr, M. Adsorption of Methanol on Si(001): Reaction Channels and Energetics. *J. Phys. Chem. C* **2018**, *122*, 14756 – 14760.

(25) Reutzel, M.; Münster, N.; Lipponer, M. A.; Länger, C.; Höfer, U.; Koert, U.; Dürr, M. Chemoselective Reactivity of Bifunctional Cyclooctynes on Si(001). *J. Phys. Chem. C* **2016**, *120*, 26284–89.

(26) Länger, C.; Heep, J.; Nikodemiak, P.; Bohamud, T.; Kirsten, P.; Höfer, U.; Koert, U.; Dürr, M. Formation of Si/organic interfaces using alkyne-functionalized cyclooctynes – precursor-mediated adsorption of linear alkynes versus direct adsorption of cyclooctyne on Si(001). *J. Phys.: Condens. Matter* **2019**, *31*, 034001.

(27) O'Donnell, K. M.; Byron, C.; Moore, G.; Thomsen, L.; Warschkow, O.; Teplyakov, A.; Schofield, S. R. Dissociation of CH_3-O as a Driving Force for Methoxyacetophenone Adsorption on Si(001). *J. Phys. Chem. C* **2019**, *123*, 22239 – 22249.
